# Implementation of Inertia Sensor and Machine Learning Technologies for Analyzing the Behavior of Individual Laying Hens

**DOI:** 10.3390/ani12050536

**Published:** 2022-02-22

**Authors:** Sayed M. Derakhshani, Matthias Overduin, Thea G. C. M. van Niekerk, Peter W. G. Groot Koerkamp

**Affiliations:** 1 Farm Technology Group, Wageningen University, 6700 AA Wageningen, The Netherlands; matthiasoverduin@hotmail.com (M.O.); peter.grootkoerkamp@wur.nl (P.W.G.G.K.); 2 Biometris, Wageningen University, 6700 AA Wageningen, The Netherlands; 3 Wageningen Livestock Research, Wageningen University, 6700 AA Wageningen, The Netherlands; thea.vanniekerk@wur.nl

**Keywords:** laying hen, daily behavior, machine learning, inertia sensor

## Abstract

**Simple Summary:**

Poultry-welfare regulations have caused a shift from cage housing towards more welfare-friendly systems with more possibilities for the birds to meet their natural behavioural needs. The welfare-friendly systems with litter allow and encourage the hens to perform natural behavior including activities that lead to increases in the amount of airborne dust particles emission from such poultry houses. For successful management of these systems, the behavior of the hens needs to be considered, which is more challenging and time-consuming for the farmer. The main objective of this study was to show a proof of principle to identify, classify and analyze the behaviors of laying hens in three levels of activity by using an inertia sensor and machine learning techniques. The model was able to predict the laying hen behaviors with an accuracy of 90%. The results of such monitoring could be used by farmers in the management of poultry houses.

**Abstract:**

Welfare-oriented regulations cause farmers worldwide to shift towards more welfare-friendly, e.g., loose housing systems such as aviaries with litter. In contrast to the traditional cage housing systems, good technical results can only be obtained if the behavior of hens is considered. With increasing flock sizes, the automation of behavioural assessment can be beneficial. This research aims to show a proof of principle of tools for analyzing laying-hen behaviors by using wearable inertia sensor technology and a machine learning model (ML). For this aim, the behaviors of hens were classified into three classes: static, semi-dynamic, and highly dynamic behavior. The activities of hens were continuously recorded on video and synchronized with the sensor signals. Two hens were equipped with sensors, one marked green and one blue, for five days to collect the data. The training data set indicated that the ML model can accurately classify the highly dynamic behaviors with a one-second time window; a four-second time window is accurate for static and semi-dynamic behaviors. The Bagged Trees model, with an overall accuracy of 89% was the best ML model with the F1-scores of 89%, 91%, and 87% for static, semi-dynamic, and highly dynamic behaviors. The Bagged Trees model also performed well in classifying the behaviors of the hen in the validation data set with an overall F1-score of 0.92 (uniform either % or decimals). This research illustrates that the combination of wearable inertia sensors and machine learning is a viable technique for analyzing the laying-hen behaviors and supporting farmers in the management of hens in loose housing systems.

## 1. Introduction

Welfare-oriented legislation, such as the European Directive [1], imposing specific regulations for the keeping of laying hens, has caused a shift from cage housing towards more welfare-friendly systems with more possibilities for the birds to meet their behavioural needs. These so-called loose housing systems typically house larger groups of hens and provide them with a litter area and more space per bird [2]. For successful management of these systems, the behavior of the hens needs to be taken into account, which makes it more challenging for the farmer and also more time-consuming [3].

The shift from cage housing to loose housing systems for laying hens substantially increased the fine-dust emissions from the poultry sector in the Netherlands, with the litter being the major additional source. Fine dust (PM10) from livestock houses consists primarily of faeces, feed and animal matter, such as hairs and feathers [4,5]. Fine dust is regarded as a pollutant that causes harmful effects for both the environment and the health and welfare of humans and animals [6,7]. The emitted PM10 (Particular Matter (PM10) with an aerodynamic equivalent diameter equal to and less than 10 µm [7]) from the poultry sector exceeds the air quality thresholds set by the European Union [8]. The type of activity of laying hens, and in particular the activities in the litter area and their intensity, have a direct and pronounced effect on PM10 emissions. Calvet et al. [9] indicated that there is a strong relationship between the concentration of fine dust in the air and the animal activity index (Ai), demonstrated in a study with three- and four-weeks old broilers. In this case, the activity index was defined as the proportion of broilers that were not laying down. The low Ai during the night and the middle of the day were caused by two dark periods during the day, in which the activity of the broilers was significantly lower.

Various techniques are being developed to reduce dust emission and they affect dust concentration in the animal house and/or in the exhaust air [10]. However, a combination of such techniques with managing bird behavior might enhance the performance of those techniques. Smart managing of light intensities and feeding times can direct birds from or towards the litter [9] and thus regulate and restrict peak dust emission. This would not only benefit the total dust emission but could also benefit the health of the human workers in the house [11].

The daily behavior of production animals needs to be monitored to apply optimal management in poultry houses. Automated monitoring of the behavior of laying hens can have advantages for managing the flock and safeguarding animal welfare [12]. The activity level and type of activity of the birds can provide useful information about individual and flock health (e.g., detect sick birds or piling) [13] and welfare status (e.g., typical positive or negative behaviors). Daily behaviors are not equally distributed over the day, therefore a good impression of behavior can only be obtained if assessed throughout the entire day. As it is too time-consuming for farmers to manually check the chicken behaviors, an automatic device can be very useful. Not all behaviors are equally important for management decisions. Therefore, chicken behaviors can be classified into different classes according to their activity levels. Kozak et al. [14] identified three main classes of laying-hen behavior based on the intensity of their individual activities. They were classified as low-, moderate- and high-intensity physical activities.

In general, animal-monitoring techniques can be divided into the following two categories: body-worn sensor technologies and remote measurement technologies. To monitor chicken behavior, both technologies have advantages and disadvantages. One of the advantages of body-worn sensor technologies is the possibility of the individual identification of chickens, as they all have their own sensor. By using remote measurement technologies, such as computer vision, the distinction between various individual animals can be more difficult. However, in the case of body-worn sensors, one sensor is needed for each chicken, which might cause problems when upscaling the system to a commercial-flock size [15].

Machine learning is a technique that is used in animal behavior analysis studies to efficiently analyze large datasets. This technique consists of a system with multiple algorithms that enable the subtraction of hidden features and relationships from datasets. The complexity of the different algorithms varies and involves several stages of sophisticated decision making, which invites the use of machine learning algorithms into optimizing automating processes [16]. These types of monitoring are even able to operate in real-time, which potentially alleviates the task of monitoring [16].

Machine learning distinguishes two main types of learning, i.e., supervised and unsupervised learning. Supervised learning consists of algorithms that attempt to classify data based on labelled input data, while unsupervised learning models a set of inputs where labelled input is not available. One supervised technique that is often applied in research is the Support Vector Machine (SVM), which is preferred because of its ability in generalization [17]. Hepworth et al. [16] showed that applying SVM techniques to recognize birds/poultry data were able to correctly predict whether a chicken was sick or not (accuracy rate of 99.5%). They also compared additional algorithms such as Bayesian classifier, Random Forest, and an artificial neural network and all these methods had accuracies above 95%.

A review of the literature reveals that there is great potential for improving the performance of these algorithms. Examples of these improvements include classifying more behavioural categories and improving the outcomes of machine learning models by validating the parameters. While accelerometers are widely used, there is no consistent approach to process the data that is being generated by these devices [16]. This restricts the ability to compare results across various research. Additionally, behavioural studies are, for practical reasons, often performed in smaller-sized systems, and results obtained by those studies do not necessarily reflect behavior in commercially sized systems [16]. The main objective of this study was to show a proof of principle of tools for analyzing laying-hen behaviors using wearable inertia sensor technology and machine learning techniques. The outcome of this research will give more insight into the feasibility of using such techniques as part of automation and management systems in the commercial poultry sector. This can aid in managing indoor air quality and dust emission.

## 2. Materials and Methods

### 2.1. Experimental Setup

An experiment was designed to collect data of the daily behavior of two individual chickens. The experiment was carried out in a section of a commercial aviary laying-hen house, which was fenced off from the rest of the house by wire mesh. The experimental area was about 5 × 4 × 4 (length-width-height) meters in size. The same facilities were available as in the rest of the house, such as a feed trough, nipple drinkers for unlimited water supply, nest boxes, perches, a pecking block, and a large litter area. The light program was similar to the rest of the house. Lights were turned on around 4:30 a.m. and turned off with a dimming phase between 7:30 p.m. and 7:45 p.m. During the experiment, 15 white laying hens were present in the experimental area. The chickens were 34 weeks of age. Two chickens were equipped with a lightweight inertial measurement unit (IMU, also called inertia sensor) of 16 g. The measurement units were mounted onto the chickens via small backpacks, consisting of a small elastic strap that was looped around the wings, together with a small bag of fabric in which the sensor was placed. Ethical approval for the experiment was granted by the Animal Welfare Body of Wageningen Research for mounting backpacks on two chickens for a maximum period of 5 days.

### 2.2. Data Acquisition and Analysis

The actual behavior of the chickens was collected simultaneously via video recordings and a wireless inertial sensor. The chicken activities were recorded during the experiment using a GoPro Hero 7 (Black) video camera. The MTw2 Awinda wireless motion tracker (IMU) of Xsens was used as the inertial measurement unit in this study. This sensor consisted of an accelerometer, a gyroscope, and a magnetometer which can measure the acceleration, angular velocity, and magnetic field, respectively. The technical properties for the IMU are provided in Table 1.

Two chickens were equipped with a backpack, in which the sensor could be fit. To be able to distinguish one from the other, the color of their backpacks differed, whereby one backpack had a green color mark and the other backpack a blue color mark. The first two days of the experiment were used to get the chickens being used to wearing backpacks. After that, no effects of the backpacks on behavior were expected [18]. During the following three days of data collection, the ‘green chicken’ wore the sensor in the first two days and the ‘blue chicken’ wore the sensor during the third day. When a chicken was not wearing a sensor, a foam dummy sensor was placed in the backpack to avoid that the chicken would behave differently in case the sensor was placed in the backpack.

As the sensor was not able to measure the time of the day, a timer was started at the same moment as the sensor was turned on. By displaying this timer in the video recordings, the time of the video and the sensor could be synchronized. The synchronization of the sensor and the video was required because the video was used for the annotation of the behaviors.

Table 2 shows the classification of the behavioural activities of laying hens based on their intensity [16]: low-, moderate- and high-intensity physical activities. Class 1 represents static laying-hen behaviors, class 2 represents semi-dynamic behaviors and class 3 represents highly dynamic laying-hen behaviors.

The classes are highly imbalanced in terms of the amount of collected data per class, as class 3 has substantially fewer data points than classes 1 and 2. By assigning higher penalties to minority classes, the ML models can equalize the weights of the classes in case of the existence of an imbalance between available data points. The penalty of misclassifying a sample of class *i* is calculated by:

(1) $$penalty_{i}=nTotal/nClass_{i}\;,$$

where *nTotal* is the total number of samples in the dataset and *nClass_i_* is the number of samples in the dataset that is annotated as class *i* [17].

The original dataset [19] consisted of the standardized dataset for the green chicken. Dataset A is a precise version of the original dataset that was redone by a trained person for class 3. Dataset B was used to analyse the classification performance of models obtained from the behavior of the blue chicken. An overview of the different datasets and their class distributions is provided in Table 3.

**Figure 1:**
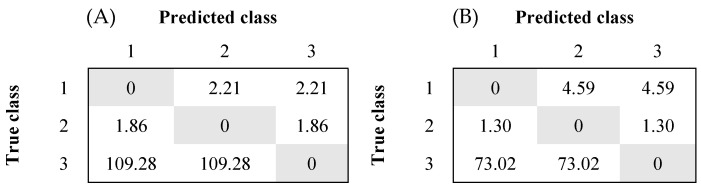
Penalty matrices were used for datasets (**A**) (left) and (**B**) (right) based on Equation (1). The rows represent the true classes, while the columns represent the predicted classes.

As seen in Figure 1, classes were highly imbalanced based on the amount of annotated data per class. Hence, penalty matrices were applied to the datasets to compensate for the effect of the imbalanced distribution of the data between these three datasets (Figure 1).

**Figure 2:**
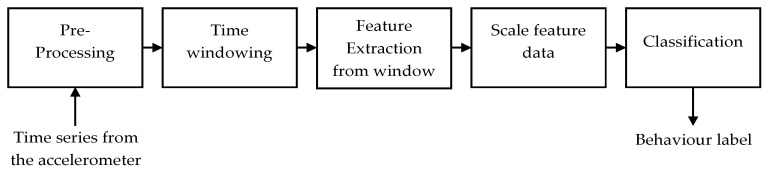
Overview of consecutive steps of the behavior classification process (adopted and modified from Figure 2 in ref [20]).

Various steps had to be completed before using the experimental data for the identification of the chicken behaviors. An overview of the main steps is shown in Figure 2. Important pre-processing steps were video annotation and linking the video data to the IMU sensor data. Raw data from the sensors were pre-processed, for which the Xsens file type changes so that it can be read by data software such as MATLAB. Time windowing was required to generalize data points and extract more information from the acceleration data. Video material was visually annotated once by trained personnel every 0.5 s. Based on the majority of types of behavior in a certain class (see Table 2), an intensity label 1, 2, or 3 scores per 0.5 s time interval. After pre-processing the IMU and annotated video data, time windows containing data of several seconds were created to combine information of multiple datapoints within that specific time window [16,17]. Two different time windows were used, namely a one-second time window and a 4 s time window. The sampling frequency of the IMU was set at 100 Hz, resulting in 100 and 400 IMU data points for the 1 and 4 s time window, respectively. Time windows were shifted 0.5 s which led to a 50% and 87.5% overlap between consecutive time windows of 1 and 4 s, respectively.

From the specific data in the windows, it was possible to calculate important characteristics, also called features [21,22,23,24]. These features could then be used for the classification of the behavior. A total of 31 features were obtained from the raw accelerometer data. The following features were extracted in X-, Y-, and Z-direction: skewness, kurtosis, mean, standard deviation, variance, minimum, maximum, entropy, energy, and covariance. In addition to these directional features, the average signal magnitude also served as a feature. Feature extraction was performed to reduce dimensionality so that the data could be classified. However, before using the feature data, the data were standardized.

Model creation and a statistical analysis were performed using MATLAB R2020b, version 1.0.0.1. (MathWorks, Inc., Natick, MA, USA) and Microsoft Office Excel 2016. The Random Forest classification model was created in MATLAB R2020b software. The Classification Learner application supported by MATLAB R2020b was used to create a basic Random Forest classification model. The Machine Learning ToolboxTM was used to analyze and model the data using different Machine Learning methods. It provided principal component analysis (PCA), regularization, dimensionality reduction, and feature selection methods that allowed identifying of features with the best predictive power. A two-tailed paired samples *t*-test was used to statistically show a significant difference between results. This was implemented to show whether, for example, a certain difference in an accuracy value would be significantly different or not.

Having a large number of features in a dataset is computationally expensive. In order to reduce the computational load, dimension-reduction techniques with a principal component analysis were applied. Principal component analysis (PCA) is a statistical method that reduces the dimensionality of the dataset by trying to find a low-dimensional representation that captures as much information as possible [25]. This technique assumes that a high variation in data is important for the model. It reduces the number of features, which is called the principal component. This research used PCA to explain at least 95% of the variance of the available data and only included features that contributed to this 95%. The first principal component is the component that explains the largest amount of variance in the data, followed by the principal component that explains the second-largest amount of variance and so on, until the desired 95% of the variance in the dataset was explained.

These data were then exposed to feature extraction, which means reducing the dimensionality, normalizing, and standardizing the dataset. The dataset was then split into a training data set and a test data set, where the training data was a subset used to train a model and the test set was used to test the trained model. Finally, the test data were used to compute the generalization performance of the model, in other words, the ability of the model to generalize the outcome.

### 2.3. Model Performance Validation

**Figure 3:**
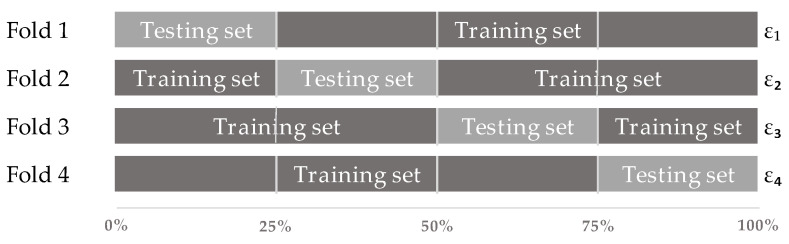
Four-fold Cross-Validation. The original dataset is partitioned into 4 equally sized subsets, which are repeatedly used as either test or training sets.

In order to assess the performance of the model, cross-validation and a principal component analysis were introduced. When training the model, overfitting and underfitting were identified and prevented. Cross-validation is a technique that splits the data in a certain way to find the best algorithm for the model. It is used to evaluate the performance of a machine learning model by predicting new datasets that the model has not previously been trained with [26]. This was performed by partitioning the available dataset, using a subset of the whole dataset to train the algorithm and the remaining data of the dataset to test the model so as to evaluate its performance. Each round involved randomly partitioning the original dataset into a training and a test set. This process was then repeated several times, as can be seen in Figure 3.

A confusion matrix was used as a validation tool. The accuracy and F1-scores were calculated to assess model performance [27,28]. The precision and recall were calculated according to:

(2) Precision=TP/(TP+FP),

(3) Recall=TP/(TP+FN),

where *TP* is the number of true positives, so where the model correctly predicts the positive class. *FP* is the number of false positives, where the model incorrectly predicts a positive class. *FN* is a false negative, where the model incorrectly predicts the negative class.

**Figure 4:**
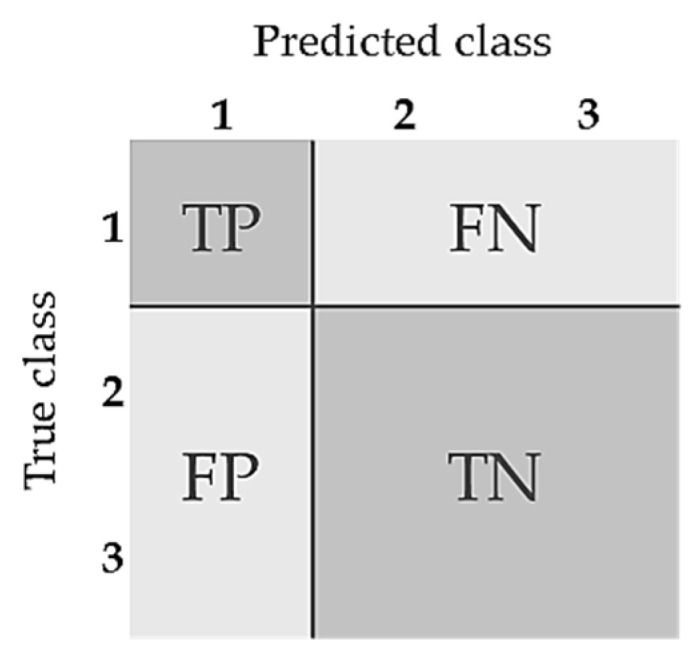
Confusion matrix of true (T), positive (P), false (F), and negative (N) labels for a dataset within a multiclass problem.

Within this research, a multiclass setting instead of a binary classification setting was used. The resulting accuracy and the F1-scores per class were therefore calculated according to:

(4) Accuracy=TP/(TP+TN+FP+FN),

(5) F1-score per class=(2×Precision×Recall)/(Precision+Recall), 

where *TP*, *FP*, and *FN* are the predictions as described above. *TN* is the outcome where the model correctly predicts the negative class. The overall F1-score was then calculated by taking the mean of the individual F1-scores of each class. Figure 4 illustrates a multiclass confusion matrix for a dataset.

## 3. Results

### 3.1. Correlation

The variables that are highly correlated with at least one other variable are the standard deviation, the variance, the minimum and maximum, and the energy for each direction (X-, Y-, and Z-direction, Pearson Correlation Coefficient > 0.7 or <−0.7).

For the 4 s time window, there was no statistical difference found between the accuracy values when all variables were included and when some highly correlating variables were removed (*p* = 0.987). However, for the 1 s time window, removing some highly correlating variables decreased the mean accuracy of all the models by 0.5% (*p* = 0.0008).

### 3.2. Principal Component Analysis

The Principal Component Analysis (PCA) was involved in the next analysis to check its effects on the performance of the different machine learning techniques. With each comparison, the classification models were trained and tested on the same datasets with the same time window, being either 1 s (short) or 4 s (long).

The effect of the randomization on cross-validation (due to randomly assigned subsets) was investigated before the PCA results were analyzed. This was performed by running the models multiple times with the same settings applied. When looking at the 1 s time window with dataset A, there was no significant difference in the accuracy values for both PCA and no PCA (*p* = 0.318 and *p* = 0.277, respectively). The 4 s time window also showed that the randomization due to applying cross-validation did not introduce significant differences with *p*-values of 0.551 and 0.341 for PCA and no PCA, respectively.

### 3.3. Time Windows

The F1-score of class 3 was not significantly different. The results are displayed in Table 4. When looking at the 4 s time window and comparing PCA vs. no PCA, not applying PCA on the original dataset resulted in a 1.5% higher accuracy value (*p* = 0.035). The overall F1-score was 2% higher (*p* = 0.0008) when no PCA was applied and the F1-scores of classes 2 and 3 were 1% and 4% higher in the case of no PCA (*p* = 0.0108 and *p* = 0.0085, respectively). The F1-score of class 1 showed no significant difference between PCA and no PCA (*p* = 0.175). Likewise, dataset A showed a 1.3% higher accuracy value when PCA was not applied (*p* = 0.0004). The F1-scores of classes 1 and 2 were both 1% higher resulting in a 3% higher overall F1-score of 0.58 (*p* = 0.0185). The F1-score of classes 1 and 2 were both 0.89 (*p* = 0.0113 and *p* = 0.0006).

The 1 s time window showed significant differences in all measured parameter values for both the original dataset and dataset A except for the F1-score of class 3 and the training time (Table 5). The original dataset had 2.7% higher accuracy values when no PCA was applied compared to the original dataset with PCA applied (*p* = 8.5 × 10^−8^). Moreover, the F1-score for class 1 was 2% higher with no PCA applied (*p* = 1.43 × 10^−9^). The F1-score for class 2 was also 3% higher in the case of no PCA (*p* = 2.32 × 10^−6^). This resulted in a 3% higher overall F1-score of 0.81 when no PCA was applied (*p* = 0.0017). Dataset A showed that not applying PCA resulted in a 2.7% higher accuracy of 83.6% versus an accuracy of 80.9% with PCA enabled (*p* = 1.49 × 10^−8^). There were also significant differences in the F1-scores of classes 1 and 2 and the overall F1-score. The F1-scores for classes 1 and 2 were 2% and 3% higher with no PCA, resulting in a 3% higher overall F1-score (*p* = 9.86 × 10^−9^, *p* = 2.20 × 10^−6^, and *p* = 0.0003, respectively). The F1-score was not significantly different as the *p*-value was equal to 0.130.

For the original dataset with no PCA applied, the overall accuracy is larger for the 4 s time window with an accuracy of 89.4%, compared to an accuracy of 85.5% for the 1 s time window (*p* = 0.0028). The F1-score of class 1 was 4% higher for the 4 s time window and the F1-score of class 2 was 3% higher (*p* = 5.58 × 10^−5^ and *p* = 1.3 × 10^−4^). The 4 s time window had a lower F1-score for class 3 with the F1-score being 0.46 versus 0.72 for the 1 s time window (*p* = 1.04 × 10^−7^). Subsequently, the overall F1-score was significantly higher for the 1 s time window with an overall F1-score of 0.80 versus an overall F1-score of 0.75 for the 4 s time window (*p* = 8.63 × 10^−5^).

For dataset A with no PCA applied, there were differences in all of the measured outcomes, except for the training time (Table 6). The models had an average accuracy of 88.8% for the 4 s time window and 83.6% for the 1 s time window (*p* = 5.48 × 10^−5^). The F1-scores for the 4 s time window were 0.89, 0.89, and 0.58, respectively, for classes 1, 2, and 3, resulting in an overall F1-score of 0.79. The 1 s time window resulted in F1-scores of 0.85, 0.82, and 0.37 for, respectively, for classes 1, 2, and 3. This resulted in an overall F1-score of 0.68. These values were statistically different with the *p*-values being 0.013, 2.67 × 10^−6^, 4.07 × 10^−7^, and 9.25 × 10^−8^, respectively, for the F1-score of class 1, 2, and 3, and the overall F1-score. Since the type of highly dynamic behavior is very short and a sharp peak in the acceleration data is often observed, the time window used is important. When time windows are longer, they contain more data of that specific event. This increases the amount of information of that event, which should increase the classification performance of the model. The results are displayed in Table 6.

The more precisely annotated labels for class 3 resulted in different performances for the different time windows with dataset A. The machine learning methods showed an increase in the F1-score of class 3 for the 4 s time window when extra data for this class was provided. However, on the 1 s time window, the F1-score showed a different result, with a lower F1-score for class 3 when extra data for this class was provided. The 4 s time window with no PCA applied showed an increase in the F1-score of class 3 from 0.46 in the original dataset to 0.58 in dataset A (*p* = 3.00 × 10^−7^). This led to an increase in the overall F1-score from 0.75 to 0.79 for, respectively, the original dataset and dataset A (*p* = 2.00 × 10^−6^). The other measured parameters such as accuracy and the F1-scores for classes 1 and 2 did not show significant differences. The 1 s time window showed a decrease of the F1-score of class 3 with 0.72 for the original dataset and 0.37 for dataset A (*p* = 1.68 × 10^−9^). This reduced the overall F1-score from 0.81 to 0.69 (*p* = 1.89 × 10^−9^).

### 3.4. Machine Learning Models

A comparison was made between an existing Random Forest model and the other available models to find the best model for this study. This means that the original dataset with a 1 s time window was selected without applying PCA. When comparing these results, the level of significance is important in order to indicate whether the results are significantly different. Therefore, two situations were investigated: one situation with the default significance level of 5% (*p* < 0.05) and one situation with a significance level of 10% (*p* < 0.1).

When looking at the default significance level, we found that there were no models significantly better than the Random Forest model. When altering the significance level to a level that allows less than 1 in 10 chance of being wrong (*p*-value of 0.1), two models showed significantly higher values. These models were the Bagged Trees and subspace KNN within the ensemble Machine Learning technique. Their *p*-values were, respectively, 0.054 and 0.097 and provided a significantly better fit on the data than the initial Random Forest model. In Random Forest, only a subset of features is selected at random, while with Bagged Trees all features are considered when splitting a node. In this research, Bagged Trees performed best. Therefore, only the results of the Bagged Trees model will be further considered.

**Figure 5:**
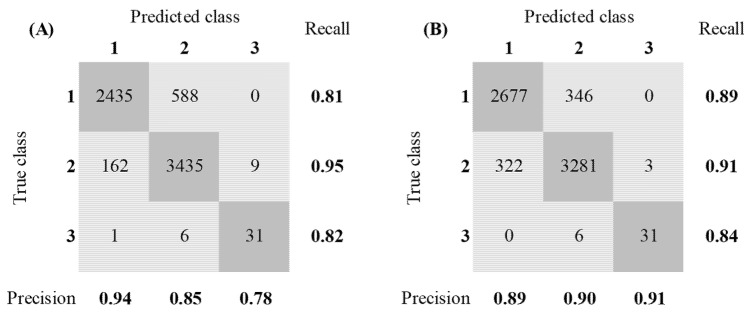
Confusion matrices of the Random Forest model (**A**) and the Bagged Trees model (**B**). (Class 1 = static behavior, class 2 = semi-dynamic behavior, class 3 = high-dynamic behavior).

The Bagged Trees model had an accuracy value of 90.0% and its F1-scores were 0.89, 0.91, and 0.87 for, respectively, classes 1, 2, and 3. This resulted in an overall F1-score of 0.89. Compared to the Random Forest model the accuracy was increased by 1% and the increase in F1-scores showed a better fit on the different classes. The F1-scores increased by 2%, 1%, and 6% for classes 1, 2, and 3, resulting in an overall F1-score increase of 3%. The confusion matrices of the Random Forest model and the Bagged Trees model are provided in Figure 5.

### 3.5. Model Validation Based on the Second Chicken (Dataset B)

**Figure 6:**
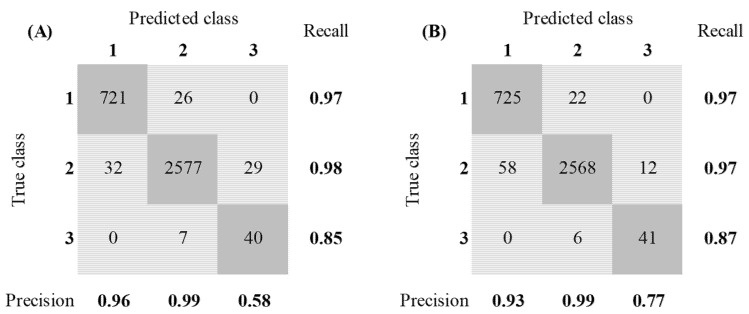
Confusion matrix of the Random Forest model (**A**) and the Bagged Trees model (**B**) with dataset B as input data and no PCA applied. (Class 1 = static behavior, class 2 = semi-dynamic behavior, class 3 = high-dynamic behavior).

To verify the classification performance of the Bagged Trees model, this model was exposed to accelerometer data and labelled data of a different chicken (blue chicken, dataset B). The confusion matrices of both models with dataset B as input data are given in Figure 6.

The same data-handling method was used to check the classification performance (short time window and no PCA applied). The Bagged Trees model was able to predict the data with an accuracy of 97.1%. This resulted in F1-scores of 0.95, 0.98, and 0.82 of classes 1, 2, and 3. The overall F1-score was equal to 0.92. Comparing these results with the results of the Random Forest model showed that the F1-score of class 1 decreased by 1% when using the Bagged Trees model instead of the Random Forest model. The F1-score of class 2 remained equal. Nonetheless, the Bagged Trees model was able to increase the F1-score of class 3 by 13%, increasing from 0.69 for the Random Forest model to 0.82 by using the Bagged Trees model. Subsequently, the overall F1-score increased by 4% (0.88 for the Random Forest model and 0.92 for the Bagged Trees model).

## 4. Discussion

### 4.1. Data Collection

Data were collected from two laying hens, from the same genotype and housed in the same environment over a limited amount of time. This could have influenced the results as other genotypes may have slightly different behavioural patterns and some environments allow more behavioural expressions than others. For this study, however, the limitations on the results are not thought to have a considerable effect. The housing system of the hens allowed them to express a normal variety of behaviors. Different genotypes, more hens and longer data recording could influence the distribution of the individual behaviors, but the classification of behaviors as used in this study will fade out small differences between breeds or caused by differences in, e.g., climate [29]. As long as these differences do not cause a completely different number of data points per behavioural class, no substantial effect is expected on the performance of the models. Therefore, the results are expected to be applicable for other housing systems and other genotypes. For application in large flocks, additional research is required to identify the minimum or the optimum number of hens that need to be equipped with sensors for reliable behavioural assessment in various parts of the henhouse.

Monitoring more than two chickens for more days might lead to more data to predict the underlying behavior of laying hens. However, because of animal-welfare concerns, it was not possible to run experiments for more than 5 days. Additionally, more data are acquired across more days, but these data will not necessarily provide information regarding the chicken behavior, therefore, the accuracy of the model will not significantly improve. In other words, it is necessary to acquire more data if one aims to identify specific behaviours of chickens while the behaviour of chickens was classified into three classes in this research and the number of chickens and the measurement days were sufficient enough for this aim. Considering the fact that the inertia sensors are relatively expensive equipment, using only two chickens to identify the chicken behaviors is one of the strengths of this research study.

### 4.2. Principal Component Analysis (PCA)

Removing highly correlating variables from the training procedure did not result in a significant improvement to model performance. This was tested to see whether the behavior of one variable could be predicted by the behavior of the other correlating variable. Initially, the non-correlated variables seemed to be independent and could help to recognize the activity level within the datasets. The only reason to remove highly correlated features would be for storage and computational load, but as these were not a problem, all variables were kept within the models. Moreover, removing the highly correlated variables resulted in decreased model accuracies. The use of Principal Component Analysis was investigated since it automatically selects a set of orthogonal Principal Components (non-correlating variables) to train the models. Application of Principal Component Analysis (PCA) did not introduce significant improvements to the model outputs. In most cases, PCA reduced the capacity of the models to correctly classify laying-hen behavior [30].

### 4.3. Cross-Validation

Besides the PCA, cross-validation can also be used as a way to prevent overfitting [31]. Since all the datasets were first exposed to cross-validation, the datasets were already evaluated based on their capacity to generalize. As the models were cross-validated using 4-fold cross-validation, the accuracy values of some scenarios were different when the models ran multiple times. As this did not show a significant difference, only a single run of all the models was considered to be part of the results. To decrease the effect of randomization during cross-validation even more, future research could run the models multiple times instead of only a single run and combine the results and take, e.g., the mean value for the accuracy. This should decrease the effect of the randomization part during cross-validation and will, therefore, increase the validity of the results of the models.

This research has shown that machine learning is able to detect laying hen behavior under different circumstances. The accuracy of the models was in all different scenarios above 70%.

As mentioned before, MATLAB used cross-validation to prevent overfitting. The selected method for this procedure was k-fold cross-validation and this includes randomization, as k-fold cross-validation partitions the data into k randomly assigned subsets of equal size [32]. This randomization caused the models to have slightly different results in some instances. In order to check the effect of the randomization on the final output of the models, the models have been run several times with the same settings applied. The results of this effect did not have a significant impact on the accuracy values of the models, so the accuracy values of one-run were considered in each scenario.

Additionally, this research focused more on the activity level of laying hens rather than the specific behaviors of laying hens. This way of classifying laying hen behavior is too general to obtain an accurate prediction of the precise laying hen behavior. Such a general prediction of laying hen behavior may be less useful for behavioural studies [18], but it may provide a useful application in commercial poultry, as commercial farmers are often more interested in flock behavior and general activity levels, rather than single specific behaviors. As a consequence, the models in this research can be beneficial in the development of precision livestock farming in the poultry sector [33].

### 4.4. Highly Dynamic Behavior

The obtained results for the high-dynamic class, class 3, show that the machine learning models were able to accurately capture this type of behavior. Due to the fact that the type of highly dynamic behavior is very short and often presents a sharp peak in the acceleration data, the time window used is important [34]. When time windows are longer, they contain more information, which increases the classification performance of the model. This was also the case when the results of the 4-s time window were compared with the 1-s time window. The 4-s time window had significantly higher accuracy values than the 1-s time window. Additionally, the F1-scores of classes 1 and 2 are in most instances significantly higher for the 4-s time window. This is due to a larger number of data points for these two classes. However, the F1-score of class 3 was significantly better when using the shorter time window. Model prediction was harder as there were fewer data points of class 3 compared to classes 1 and 2. As fewer data provide the model with fewer data to make correct predictions. Due to the low amount of data points of class 3, a 4 s time window will generally have fewer assigned data points of this class compared to a 1 s time window. This explains the difficulty of correctly predicting class 3 with a 4 s time window [14,34].

When highly dynamic behavior is of greater interest than static behavior, a shorter time window will lead to higher F1-scores and is, therefore, more favourable than a longer time window. However, using a shorter time window is at the expense of having lower classification performance on static- and semi-dynamic behavior.

As shown by Calvet et al. [9], there is a relationship between the behavior of chickens and dust concentration inside the indoor air, as also observed by Winkel [4]. The obtained results of this research indicated that the highly dynamic behaviors such as scratching/dust bathing can be determined with acceptable accuracy even with a few data sets. Distinguishing the chicken behavior can help to identify the distribution of different dynamic behavior in time or its spread out over the day. Consequently, this information can be used in air-quality control and reduction of indoor dust concentration and fine dust emission into the environment (e.g., temporarily lower/higher ventilation rates, or applying fine-dust-reduction techniques more intensively) [11].

### 4.5. Performance of the ML Models

The largest improvement of using the Bagged Trees model over a Random Forest model was found in the classification performance of highly dynamic behaviors. Both the Bagged Trees model and the Random Forest model draw random bootstrap samples from the training set. However, besides the bootstrap samples, the Random Forest model draws random subsets of features for training the individual trees, while in bagging the full set of features is provided to each tree. This random feature selection in the Random Forest causes the trees to become more independent of each other compared to regular bagging. This research has shown that this random feature selection causes a worse classification performance for highly dynamic behaviors, while not significantly impacting the performance of the model on static- and semi-dynamic behaviors. Therefore, the regular bagged tree model is preferred over the Random Forest model [35].

### 4.6. Sensor Technology

There are multiple inertial sensors available. The main distinction is made between high-end inertial sensors and low-cost inertia sensors or custom-made inertial sensors. Two types of high-end inertial sensors are the Xsens IMU, which was used in this study, and the Physilog 5. These sensors distinguish themselves from the low-cost sensors due to their ability to measure the 3D acceleration, the 3D rate of turn, and the 3D magnetic field all in one device. Additionally, data are easily transferrable with USB, and they are water and dust resistant with IP67/IP64 certification.

Some examples of low-cost inertial sensors are the Sparkfun, the WitMotion, and Zstar3. The limitations of low-cost sensors are often that they suffer from poor signal to noise ratios and limited dynamic ranges. Nandy et al. [36] provided a way to produce a custom-made inertial sensor.

## 5. Conclusions

The novelty of this study was that it proved the feasibility of using machine learning models and inertia sensors to identify and classify laying-hen activity at various levels. We showed a proof of principle of tools for analyzing laying-hen behaviors by using wearable inertia sensor technology and machine learning models (ML).

The machine learning models of this study can predict the three activity levels of laying hens with overall accuracy values of over 90%. Removing highly correlating variables did not introduce significant model improvement with the original dataset. Additionally, applying PCA did not result in better model classification performance. Static behaviour and semi-dynamic behaviour can best be analysed with a long time window, whereas highly dynamic behaviour favours a short time window. Annotating the data of class 3 more precisely only increased model performance for the long time window. The best performing machine learning model was the bagged tree model with an accuracy of 90% for the original dataset.

The methodology developed in this paper can be used in the development of precision livestock farming systems for the commercial poultry sector. The outcomes of this research show that machine learning is able to accurately analyse different activity levels of laying-hen behaviour. This provides a reason for further research to analyse laying-hen behaviour in more detail in the future.

## Figures and Tables

**Table 1:** Technical properties of the MTw2 Awinda Wireless 3DOF Motion Tracker (www.xsens.com, accessed on 8 April 2020).

Parameter	Angular Velocity	Acceleration	Magnetic Field
Dimensions	3 axes	3 axes	3 axes
Full scale	2000 deg/s	160 m/s^2^	1.9 Gauss
Non-linearity	0.1% of FS	0.5% of FS	0.1% of FS
Bias stability	10 deg/hr	0.1 mg	-
Noise	0.01 deg/s/√Hz	0.01 µg/√Hz	0.2 mGauss/√Hz
Alignment error	0.1 deg	0.1 deg	0.1 deg
Bandwidth	180 Hz	180 Hz	10–60 Hz (var.)

**Table 2:** Classification of physical activity of laying hens based on their intensity (adopted and modified from Table 2 in ref [14]).

Class 1	Class 2	Class 3
Low-intensity	Moderate-intensity	High-intensity
• Sleep like resting • Neck shortening resting • Sleeping • Quiet sitting/standing • Small postural head/shoulder/neck movements • Perching • Egg laying • Side-laying phase of dust bathing	• Preening • Foraging & pecking • Drinking & eating • Small wing adjustments • Scratching & stretching • Head shaking • Feather fluffing • Searching behavior • Scratching behavior of dust bathing	• Walking • Running • Jumping • Wing flapping • Controlled aerial ascent/descent • Full-body shaking • Shaking phase of dust bathing

**Table 3:** Dataset overview with the available number of data points per class and additional information. Two chickens were observed, one wearing a green backpack and one wearing a blue backpack.

	Original Dataset	Dataset A	Dataset B
Number of datapoints class 1	3023	3017	747
Number of datapoints class 2	3606	3588	2638
Number of datapoints class 3	37	61	47
Total number of data points	6666	6666	3432
Chicken (color)	Green	Green	Blue
Day of recording	Wednesday	Wednesday	Friday
Total length of the recording	2 h 20 min	2 h 20 min	29 min

**Table 4:** Overview of the results for the 4 s time window for the original dataset and dataset A. Here, a comparison was made between applying Principal Component Analysis (PCA) or not applying PCA (No PCA). The *p*-value is provided to show whether the two values are significantly different (*p*-value < α, *p*-value < 0.05) or not. Significance was tested with a paired samples *t*-test with a two-tailed distribution.

Parameter	Original Dataset			Dataset A		
	PCA	No PCA	*p*-Value	PCA	No PCA	*p*-Value
Accuracy	87.9	89.4	0.0358	87.5	88.8	4.32 × 10^−4^
F1-score class 1	0.88	0.89	0.175	0.88	0.89	0.0113
F1-score class 2	0.88	0.89	0.0108	0.88	0.89	5.54 × 10^−4^
F1-score class 3	0.42	0.46	8.52 × 10^−3^	0.55	0.58	0.214
Overall F1-score	0.73	0.75	7.66 × 10^−4^	0.77	0.79	0.0185

**Table 5:** Overview of the results for the 1 s time window for the original dataset and dataset A. Here, a comparison was made between applying Principal Component Analysis (PCA) or not applying Principal Component Analysis (No PCA). The *p*-value is provided to indicate whether the two values are significantly different (*p*-value < α, *p*-value < 0.05) or not. Significance was tested with a paired samples *t*-test with a two-tailed distribution.

Parameter	Original Dataset			Dataset A		
	PCA	No PCA	*p*-Value	PCA	No PCA	*p*-Value
Accuracy	82.8	85.5	8.52 × 10^−8^	80.9	83.6	1.49 × 10^−8^
F1-score class 1	0.83	0.85	1.40 × 10^−9^	0.83	0.85	9.90 × 10^−7^
F1-score class 2	0.83	0.86	2.30 × 10^−6^	0.81	0.84	2.20 × 10^−6^
F1-score class 3	0.67	0.72	0.0780	0.34	0.37	0.130
Overall F1-score	0.78	0.81	1.67 × 10^−3^	0.66	0.69	3.10 × 10^−4^

**Table 6:** Overview of the results for the long time window (4 s, called Long) and the short time window (1 s, called Short) for datasets original and A. No PCA was applied. The *p*-value is provided to indicate whether the two values are significantly different (*p*-value < α, *p*-value < 0.05) or not. Significance was tested with a paired samples *t*-test with a two-tailed distribution.

Parameter	Original Dataset			Dataset A		
	Long	Short	*p*-Value	Long	Short	*p*-Value
Accuracy	89.4	85.5	2.82 × 10^−4^	88.8	83.6	5.48 × 10^−5^
F1-score class 1	0.89	0.85	5.58 × 10^−5^	0.89	0.85	1.31 × 10^−2^
F1-score class 2	0.89	0.86	1.31 × 10^−4^	0.89	0.84	2.67 × 10^−6^
F1-score class 3	0.46	0.72	1.04 × 10^−7^	0.58	0.37	4.07 × 10^−7^
Overall F1-score	0.75	0.81	8.63 × 10^−5^	0.79	0.69	9.25 × 10^−8^

## Data Availability

The data presented in this study are available on request from the corresponding author. The data are not publicly available because they contain information that could compromise the privacy of the farmer.
